# Management of Humeral Solitary Bone Cysts in Children Using Elastic Stable Intramedullary Nailing—A Safe and Effective Minimally Invasive Treatment

**DOI:** 10.3390/children13040537

**Published:** 2026-04-13

**Authors:** Zoltán Derzsi, Evelyn Kovács, Arpad Solyom, Corneliu-Florin Buicu, Zsolt Bara, Dávid Keresztesi-Kis, Emőke Horváth, Tibor Mezei, Réka Sólyom, Horea Gozar

**Affiliations:** 1 Department of Pediatric Surgery and Orthopedics, George Emil Palade University of Medicine, Pharmacy, Science and Technology of Targu Mures, 38 Gheorghe Marinescu Street, 540142 Targu Mures, Romania; zoltan.derzsi@umfst.ro (Z.D.); zsolt.bara@umfst.ro (Z.B.); horea.gozar@umfst.ro (H.G.); 2 Clinic of Pediatric Surgery and Orthopedics, County Emergency Clinical Hospital Targu Mures, 50 Gheorghe Marinescu Street, 540136 Targu Mures, Romania; keresztesikisdavid@gmail.com; 3 Department of Orthopedics-Traumatology, George Emil Palade University of Medicine, Pharmacy, Science and Technology of Targu Mures, 38 Gheorghe Marinescu Street, 540142 Targu Mures, Romania; arpad.solyom@umfst.ro; 4 Clinic of Orthopedics and Traumatology, County Emergency Clinical Hospital of Targu Mures, 50 Gheorghe Marinescu Street, 540136 Targu Mures, Romania; 5 Department of Public Health and Management, George Emil Palade University of Medicine, Pharmacy, Science and Technology of Targu Mures, 38 Gheorghe Marinescu Street, 540142 Targu Mures, Romania; florin.buicu@umfst.ro; 6 Department of Pathology, Faculty of Medicine, George Emil Palade University of Medicine, Pharmacy, Science and Technology of Targu Mures, 38 Gheorghe Marinescu Street, 540142 Targu Mures, Romania; emoke.horvath@umfst.ro (E.H.); tmezei@pathologia.ro (T.M.); 7 Pathology Service, County Emergency Clinical Hospital of Targu Mures, 50 Gheorghe Marinescu Street, 540136 Targu Mures, Romania; 8 Department of Pediatrics II, George Emil Palade University of Medicine, Pharmacy, Science and Technology of Targu Mures, 38 Gheorghe Marinescu Street, 540136 Targu Mures, Romania; reka.solyom@umfst.ro; 9 Clinic of Pediatrics II, 42 Gheorghe Marinescu Street, 540139 Targu Mures, Romania

**Keywords:** solitary bone cyst, pediatric orthopedics, humerus, elastic stable intramedullary nailing

## Abstract

Background/Objectives: The radiological and clinical outcomes of elastic stable intramedullary nailing (ESIN) for pediatric humeral solitary bone cysts (SBCs) were assessed, with particular attention to healing, recurrence, complications, and functional recovery. Methods: This retrospective institutional study included 25 children with humeral solitary bone cysts treated with elastic stable intramedullary nailing between January 2019 and December 2025. Patients treated exclusively with ESIN and with a minimum follow-up of 12 months were included. Radiographic healing was assessed using the Capanna classification. Clinical outcomes included pain, range of motion, complications, refracture, and recurrence. Results: The cohort consisted of 16 boys (64%) and 9 girls (36%), with a median age of 11 years. Pathological fractures were present in 56% of cases. A total of 29 surgical procedures were performed, including growth-related implant exchanges in selected patients. Cyst dimensions decreased progressively during follow-up (*p* < 0.001). At final evaluation, 64% of lesions demonstrated complete healing (Capanna I), 28% incomplete healing (Capanna II), and 8% recurrence (Capanna III); no cases of non-response were observed. No recurrent fractures or postoperative complications occurred. Pain resolved within one month in all patients, and a full range of motion was restored. Conclusions: Elastic stable intramedullary nailing appears to provide reliable stabilization and promotes cyst healing in pediatric humeral SBCs, with low recurrence rates and excellent functional outcomes.

## 1. Introduction

Solitary bone cysts (SBCs), also known as unicameral bone cysts, are common benign, fluid-filled lesions that primarily affect growing children [1].

The precise etiology remains uncertain; proposed mechanisms include disturbances in venous drainage and abnormalities in bone growth [2,3]. Recent molecular studies have identified characteristic EWSR1/FUS-NFATC2 gene rearrangements in a subset of cases [4]. Histopathologically, the cavity of a simple bone cyst is lined by a thin fibrous membrane, which may contain cementum-like structures (immature calcified and mature bone structures), reactive cells and inflammatory mediators [5,6].

Unicameral bone cysts occur from early childhood through the second decade with peak incidence between 8 and 14 years of age [7] and show a pronounced male predominance, with an approximate male-to-female ratio of 2:1 [8]. Although often asymptomatic, the main concern is the risk of pathological fractures due to cortical thinning, which may present with pain, swelling, or deformity [2,9].

Approximately 80–90% of SBCs involve the proximal humerus, followed by the proximal femur and tibia [8]. Based on their proximity to the growth plate, cysts are classified as active, within 10 mm of the physis, with continued growth potential or latent, displaced toward the diaphysis, characterized by reduced activity [9].

Solitary bone cysts may demonstrate a tendency toward spontaneous resolution over time, particularly following skeletal maturation or after pathological fracture, which should be taken into account when evaluating treatment outcomes [10,11]. Several treatment options have been described in the literature, such as observation, curettage with bone grafting, autologous bone grafting, intralesional steroid injections, intracyst injection of demineralized bone matrix, and decompression using a cannulated screw. However, these techniques are associated with variable outcomes [12,13,14].

In recent years, elastic stable intramedullary nailing (ESIN) has emerged as a minimally invasive alternative, offering mechanical stability, internal decompression of the cyst cavity, and early mobilization [15]. Many authors have reported that ESIN promotes cyst healing while reducing recurrence rates [11,16].

At our institution, ESIN has been routinely implemented since 2015.

The aim of this study was to evaluate radiological healing, healing time, recurrence rate, complications, and overall cyst resolution following ESIN treatment.

## 2. Materials and Methods

This study was designed as a retrospective observational institutional study based on clinical and radiological data, including 25 pediatric patients diagnosed with SBCs of the humerus who underwent surgical treatment using elastic stable intramedullary nailing (ESIN) between January 2019 and December 2025 at our institution.

Although humeral bone cysts have also been managed in our clinic using conventional methods, 22 patients treated with these techniques were excluded to ensure a homogeneous study cohort.

Inclusion criteria were: patients under 18 years of age with imaging and clinically confirmed diagnosis, treatment performed exclusively using ESIN technique, and a minimum follow-up duration of 12 months after surgery.

Exclusion criteria were: cysts located at other anatomical sites, patients lost to follow-up, management with other techniques, and incomplete clinical or imaging documentation.

The study was conducted in accordance with the Declaration of Helsinki and approved by the Ethics Committee of the hospital (Comisia de Etica Medicala pentru Studiul Clinic al Medicamentului din cadrul Spitalului Clinic Judetean de Urgenta Targu Mures, approval no. Ad.346650). Informed consent was obtained from all patients and/or their legal guardians.

### 2.1. ESIN Surgical Technique

At our institution, surgical intervention was performed in cases of large solitary bone cysts. In patients with pathological fractures, the treatment strategy depended on fracture displacement: displaced fractures were managed surgically at presentation, whereas non-displaced fractures were initially treated conservatively, with close radiological follow-up to assess potential spontaneous cyst healing. If no evidence of healing was observed after approximately 3 months, surgical intervention using elastic stable intramedullary nailing was performed.

Plain radiographs were obtained in all patients, while CT or MRI was used selectively in cases of complex pathological fractures or when further assessment was required to rule out vascular or neurologic compromise. In addition to imaging and clinical evaluation, laboratory investigations were performed in all patients, including complete blood count, inflammatory markers, basic biochemical tests, and tumor marker analysis.

All surgeries were performed under general anesthesia and fluoroscopic guidance. In patients with pathological fractures, closed reduction was achieved before nail insertion.

Accurate determination of the diameter of each intramedullary nail was essential for optimal stability. The nail diameter was selected to be approximately 25–30% of the medullary canal diameter. Typically, 2.0–3.0 mm elastic titanium nails were used.

For the retrograde approach (in proximal metaphyseal, proximal third, and middle-third shaft humeral fractures), the skin incision was started approximately 1 cm proximal to the lateral epicondyle and extended about 3–4 cm. The first entry point was made in the proximal end of the incision, and the second was positioned about 1–2 cm distally and 0.5–1 cm medially from the first one.

For the anterograde approach (in distal third fractures), the entry point was established just below the insertion of the deltoid muscle. If the deltoid insertion was not clearly palpable, the entry point was placed at least 4–5 cm distal to the proximal humeral physis (depending on the child’s size) and marked on the skin. The second entry point was located approximately 2 cm distal and 1 cm anterior to the first.

In both approaches, a 3–4 cm incision was performed, followed by fascial and blunt separation of muscle fibers to expose the bone surface. The cortical bone was perforated using an awl, and correct positioning was confirmed fluoroscopically.

After pre-bending, the nails were introduced sequentially into the medullary canal and advanced towards the cystic cavity under continuous fluoroscopic guidance. In cases of pathological fractures, a closed fracture reduction was performed as well. The nails were adjusted to the appropriate length in order to achieve adequate stability and also prevent soft-tissue irritation. In our technique, gentle debridement of the cyst cavity was performed using the tip of the nail. This mechanical disruption of the cyst lining may enhance healing by reducing cystic fluid production and stimulating bone formation.

Postoperatively, patients with pathological fractures were immobilized using a Desault’s bandage for two weeks, followed by two weeks of orthotic bracing.

Return to sports and full physical activity was permitted only after radiographic confirmation of cortical consolidation and cyst healing.

### 2.2. Postoperative Evaluation and Follow-Up

Standard anteroposterior (AP) and lateral (LL) radiographs of the affected humerus were obtained on the first postoperative day and subsequently at 1, 6, 12, and 24 months after surgery. Annual follow-ups were performed thereafter to evaluate long-term outcomes.

Radiologic assessment of healing was performed according to the Capanna classification, which distinguishes four grades [12]:

- Grade 1: Complete healing: full ossification of the cyst with no residual cystic area.
- Grade 2: Partial, incomplete healing: small residual cystic area persists.
- Grade 3: Recurrence: reappearance of radiolucent areas in a previously healed cyst, associated with cortical thinning.
- Grade 4: No response: persistence of cyst without radiologic progression toward healing.

At each follow-up visit, clinical evaluation included assessment of pain, range of motion of adjacent joints, and any signs of refracture or postoperative complications.

Healing was defined by cortical thickening visible on both AP and LL radiographs, together with progressive consolidation of the cystic cavity. The time to healing was measured from the date of the surgery to the first radiographic appearance of these healing criteria.

Recurrence was defined as the reappearance or enlargement of a radiolucent cavity after initial radiographic healing, corresponding to Capanna Grade 3 changes.

Functional outcome was systematically evaluated at each follow-up visit. Pain intensity was quantified using an age-appropriate visual analog scale (VAS), and the range of motion (ROM) of the shoulder and elbow was assessed using standard clinical examination.

Follow-up evaluation also included refracture, nail prominence or soft-tissue irritation, infection, and neurovascular symptoms.

## 3. Results

Two patients were lost to follow-up after the 12-month evaluation and were excluded from the final analysis.

Therefore, the final cohort consisted of 25 patients (16 boys, 64%; 9 girls, 36%) with solitary bone cysts (SBCs) of the humerus who underwent ESIN treatment at our institution between 2019 and 2025. Baseline demographic and clinical characteristics are summarized in Table 1. Overall, 29 surgical procedures were performed due to growth-related implant exchanges in selected cases. The median age at surgery was 11 years (range 6–17 years). The right humerus was affected in 14 cases (56%) and the left in 11 cases (44%). Cyst localization was proximal metaphyseal in 19 lesions (76%), diaphyseal in 5 (20%), and distal metaphyseal in 1 (4%). No significant age difference between sexes was observed (the median age was 12 years for boys and 10 years for girls, *p* = 0.15), and fracture occurrence did not differ between right- and left-sided lesions (57% vs. 55%, *p* = 0.91).

**Figure 1:**
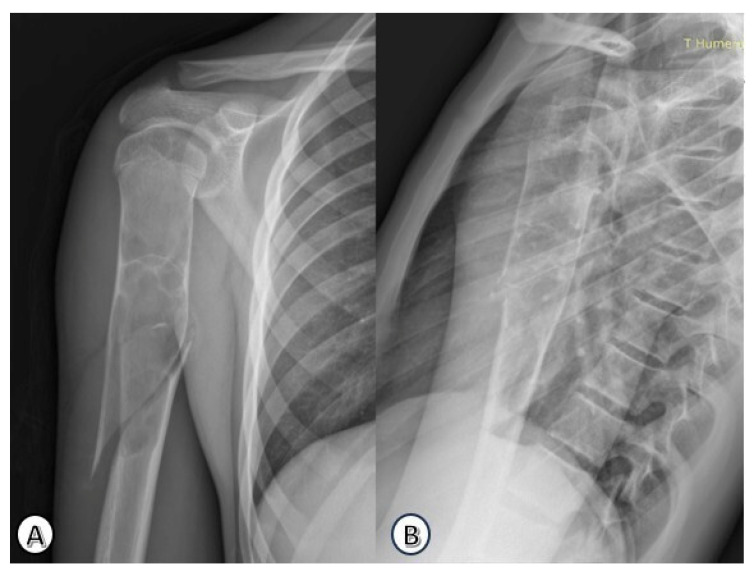
Preoperative anteroposterior (**A**) and lateral (**B**) radiographs of the right humerus revealing a simple bone cyst with an associated pathological spiral fracture in a 16-year-old male patient.

**Figure 2:**
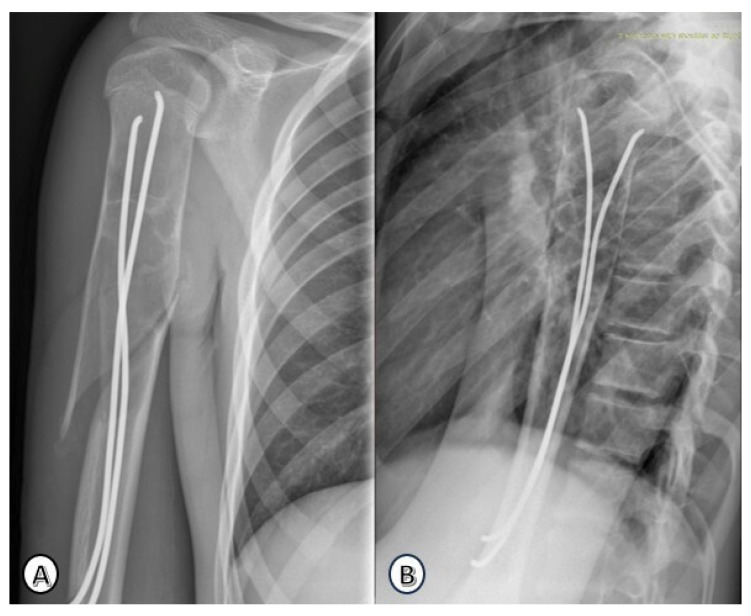
Postoperative radiographs of the same patient after intramedullary stabilization using a 3 mm titanium elastic nail. (**A**): Postopertaive anteroposterior view, (**B**): Postopertaive lateral view.

The most common presenting symptom was pain (15/25; 60%), followed by swelling (5/25; 20%) and deformity (5/25; 20%). Pathological fractures were present at diagnosis in 14 patients (56%), all of which were closed fractures. A representative case of a pathological fracture is shown in Figure 1, with subsequent stabilization using elastic stable intramedullary nailing illustrated in Figure 2.

Active cysts were identified in 17 patients (68%), whereas 8 lesions (32%) were classified as latent. No recurrent fractures occurred during follow-up.

The median hospitalization duration was 4 days (range 1–5). Patients with pathological fractures had significantly longer hospital stays compared to those without fractures (median 4 vs. 2 days, Mann–Whitney U, *p* < 0.05).

**Figure 3:**
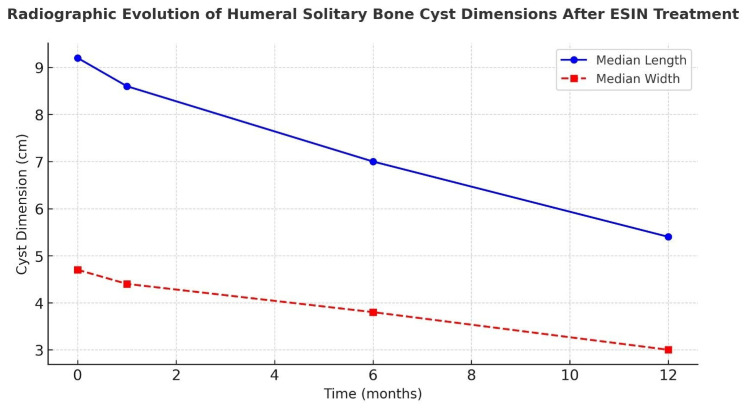
Radiographic evolution of median cyst dimensions (length and width) after ESIN treatment for humeral solitary bone cysts, demonstrating progressive reduction at 1, 6, and 12 months postoperatively.

Radiographic measurements demonstrated a progressive reduction in cyst dimensions over time. Both cyst length and width decreased significantly across the 1-, 6-, and 12-month follow-up intervals (Friedman test, *p* < 0.001), as shown in Figure 3. Post hoc Wilcoxon signed-rank tests confirmed that cyst dimensions at 12 months were significantly smaller than baseline, 1-month, and 6-month measurements (all *p* < 0.05), demonstrating continuous radiological regression (Figure 3).

Radiological evaluation using the Capanna classification showed that most patients achieved Capanna stage I (complete healing) or stage II (incomplete healing with residual radiolucency), while stage III was observed in only a few cases following intramedullary stabilization. At final follow-up, 16 lesions (64%) were Stage I, 7 (28%) were Stage II, and 2 (8%) were Stage III; no Stage IV lesions were identified. The median healing time was 15 months for Stage I and 20 months for Stage II. The median follow-up was 48 months for Stage I, 50 months for Stage II, and 54 months for Stage III, as detailed in Table 2.

**Figure 4:**
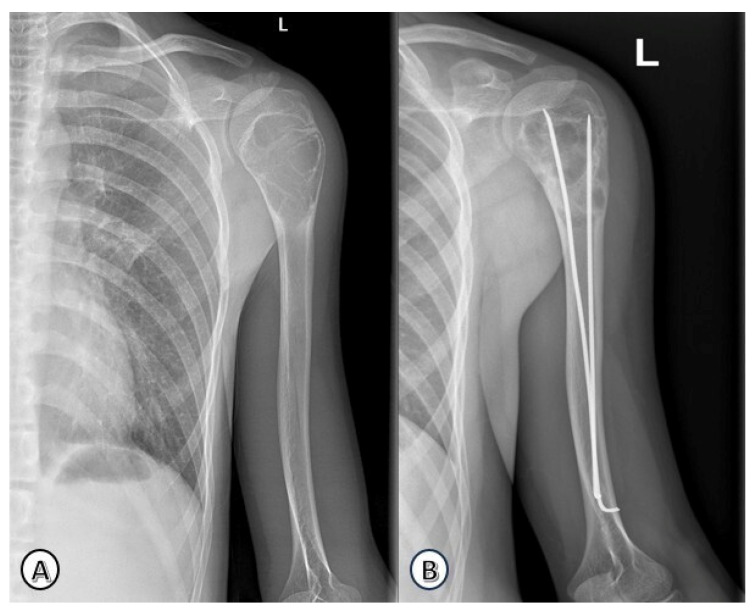
Radiographic evolution of a humeral solitary bone cyst in a 15-year-old boy. (**A**) Preoperative anteroposterior view of the left proximal humeral metaphysis showing a cystic lesion. (**B**) Follow-up radiograph at 1-year post-treatment with ESIN, demonstrating gradual remodeling and cortical thickening.

**Figure 5:**
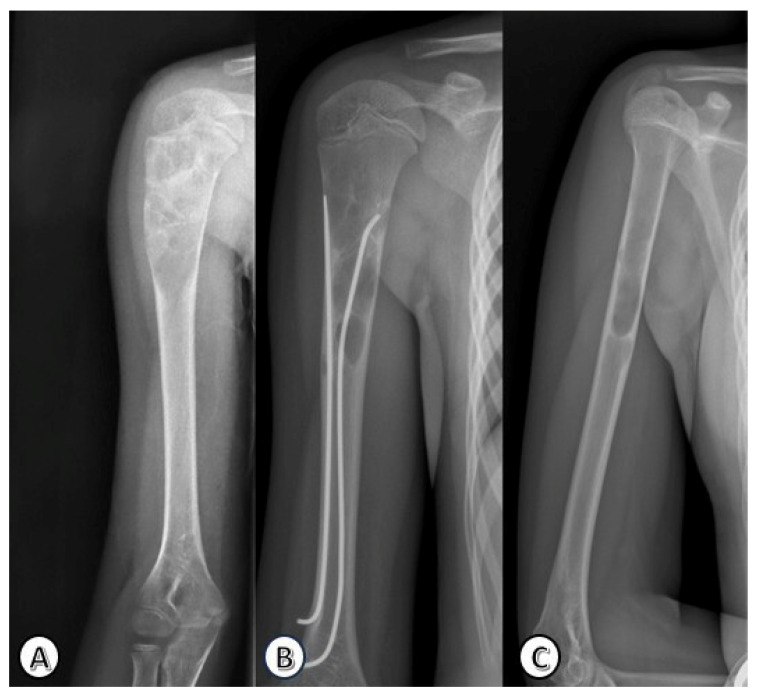
Radiographic progression of a humeral solitary bone cyst in a 15-year-old girl involving the right proximal meta-diaphysis. (**A**) Preoperative anteroposterior view showing the cystic lesion. (**B**) Radiograph after ESIN fixation. (**C**) Follow-up radiograph after ESIN removal, demonstrating complete cyst resolution and bone remodeling.

Radiographic progression and remodeling following ESIN are illustrated in Figure 4 and Figure 5.

Two cyst recurrences (8%) were identified during follow-up, both occurring 6–8 months after TEN removal. No secondary intervention was performed; both cases were managed with continued observation. One lesion subsequently resolved completely, whereas the other persisted as a small residual cyst of approximately 1 cm without pain or functional limitation.

During follow-up, 4 of the 25 patients required ESIN exchange due to rapid growth (1 girl and 3 boys). In these patients, implant exchange was required due to significant skeletal growth during follow-up, which resulted in the ESIN becoming relatively short and less effective in maintaining adequate mechanical stability. In these cases, suboptimal cyst healing was also observed. The original implant was replaced with a larger-diameter implant after a growth spurt, without associated pain, functional limitation, or implant-related complications. ESINs were electively removed at skeletal maturity (median age 18 years), since none of the patients developed implant-related pain, irritation, or sport-limiting symptoms prior to removal.

Pain improved rapidly postoperatively and had resolved in all patients by 1 month (VAS 0). Limited ROM was observed at 1 month exclusively in patients with pathological fractures; however, all patients recovered a full range of motion by around 3 months. No persistent functional impairment was noted.

No postoperative complications occurred. Specifically, there were no cases of refracture, nail prominence or irritation, infection, neurovascular symptoms, or cyst persistence.

## 4. Discussion

In the present observational study, outcomes were achieved using ESIN as a standalone technique, without adjunctive cyst-directed interventions. This observation supports the concept that intramedullary stabilization alone may provide sufficient mechanical and biological conditions to promote cyst resolution.

Although pathological fractures of the upper extremity are frequently managed non-operatively [6], this strategy may not be adequate in all cases. Surgical treatment was performed in displaced or recurrent fractures, large cysts with significant cortical thinning, and lesions with high risk of refracture or in children who engage in high levels of physical activity or sports.

One of the most relevant findings of our study is the complete absence of recurrent fractures during follow-up, despite the fact that more than half of the patients presented with pathological fractures at diagnosis. Comparable outcomes have been reported in an ESIN-only series by Knorr, Schmittenbecher and Dietz, in which intramedullary fixation of humeral cysts resulted in reliable healing without refracture events [17]. Similar observations have been reported in prospective series evaluating preventive flexible intramedullary nailing for high-risk proximal humeral simple bone cysts, in which intramedullary stabilization was associated with reliable cyst healing and the absence of fracture-related complications [1].

Reported complication rates for pediatric ESIN are low, usually involving minor skin irritation or superficial wound-related issues [10]. In line with the existing literature, no wound complications, infections, or nerve injuries were observed in our cohort.

The 96% healing rate (24/25) observed in our cohort exceeds previously reported rates for ESIN-only treatment, where healing of approximately 70% and recurrence of <10% have been documented [9], suggesting that fracture prevention and controlled cyst remodeling under intramedullary support may enhance cyst resolution.

The optimal timing for intramedullary nailing removal remains controversial. Several authors advocate early removal, suggesting that retrieval becomes technically more demanding after cyst healing and cortical remodeling and that prolonged retention may increase the risk of nail burial within the cortex. Zhang et al. likewise recommended removal within 1–2 years postoperatively [10]. In contrast, other surgeons prefer delayed removal after puberty or skeletal maturity, based on the hypothesis that prolonged stabilization may reduce refracture risk and allow for more complete remodeling. In our cohort, ESINs were electively removed at skeletal maturity, and no implant-related pain, irritation, or sports limitation was observed prior to removal. Despite this delayed approach, two recurrences were detected after TEN removal, as detailed above. Growth-related implant exchange has also been reported in the literature. Roposch et al. [18] described 32 patients treated with flexible intramedullary nails for unicameral bone cysts of long bones, in whom 9 implants required exchange due to longitudinal growth, and 2 recurrences were observed during follow-up [19]. Similarly, in our study, 4 patients underwent ESIN exchange following a growth spurt. Although exchange represents an additional procedure, it may prevent premature implant removal in skeletally immature patients and reduce the theoretical risk of recurrence during the remodeling phase.

Given the limited evidence and heterogeneous removal strategies across studies, larger cohorts and prospective comparative analyses are warranted to clarify whether implant retention duration influences recurrence risk or radiological healing trajectories.

The internal drainage effect created by the intramedullary nails may contribute to this favorable healing profile by reducing intracystic pressure and allowing gradual ossification. This mechanism has been proposed previously: when elastic intramedullary nails bridge the cyst cavity, they provide both mechanical support and continuous decompression, facilitating cyst healing and early mobilization [20]. The mechanical effect of the nail tip on the cyst lining may contribute to healing by facilitating intracystic decompression and promoting a favorable osteogenic environment. Similar mechanisms have been described in the literature, where the tip of the elastic nail is reported to disrupt the inner wall of the cyst and promote an osteogenic environment [18,21]. Furthermore, the importance of mechanical disruption of the cyst membrane in achieving healing has also been emphasized in earlier studies [22,23].

Functional outcomes were excellent in our patient profile. Pain resolved in all patients by the 1-month follow-up, and range-of-motion limitations were only transient, occurring exclusively in patients with pathological fractures. All children regained full shoulder and elbow mobility thereafter, and no long-term functional deficits were noted. Favorable functional outcomes have been demonstrated in similar cohorts, supporting the utility of ESIN in pediatric SBCs [18,24].

This study has some limitations. Its retrospective design and relatively small sample size may limit the generalizability of the findings. The lack of a comparison group treated with alternative methods also restricts direct comparative conclusions.

## 5. Conclusions

In conclusion, our findings suggest that ESIN appears to provide adequate mechanical stabilization and is associated with favorable cyst healing in pediatric humeral simple bone cysts. In our series, the absence of recurrent fractures and the generally favorable functional and radiological outcomes indicate that intramedullary fixation may represent a safe and minimally invasive treatment option in selected cases. These results support the potential role of ESIN in maintaining the structural stability of the humerus during growth and in facilitating early mobilization and return to normal activities in active children. However, given the absence of a comparative group, these findings should be interpreted with caution.

Further prospective and comparative studies with larger cohorts are warranted to confirm these observations and to better define optimal treatment strategies.

## Figures and Tables

**Table 1:** Baseline demographic, clinical, and cyst characteristics of pediatric patients with humeral solitary bone cysts treated with ESIN.

Variable	N (%) or Median [Range]
Total patients	25
Sex	16 M (64%)
	9 F (36%)
Age (years)	11 years [6–17]
Affected side	14 right (56%)
	11 left (44%)
Localization	19 proximal metaphyseal (76%)
	5 diaphyseal (20%)
	1 distal metaphyseal (4%)
Cyst dimension	
median cyst height (mm)	68.1
median cyst width (mm)	20.2

**Table 2:** Distribution of patients according to Capanna classification of humeral solitary bone cysts, including number of cases, median healing time, and median follow-up duration.

Capanna Stage	Number of Patients (%)	Median Healing Time (Months)	Median Follow-Up (Months)
I	16 (64%)	15	48
II	7 (28%)	20	50
III	2 (8%) *	-	54
IV	0	-	-

* Both recurrences occurred after ESIN removal; one resolved spontaneously during follow-up, while the other persisted as a small asymptomatic residual cyst.

## Data Availability

The data presented in this study are available on request from the corresponding author due to privacy and ethical restrictions related to patient data.

## Abbreviations

The following abbreviations are used in this manuscript:

AP	Anteroposterior view
ESIN	elastic stable intramedullary nail
LL	lateral view
ROM	range of motion
SBC	simple bone cyst
VAS	visual analog score
